# Size-dependent filtration efficiencies of face masks and respirators for removing SARS-CoV-2–laden aerosols

**DOI:** 10.1017/ice.2020.366

**Published:** 2020-07-23

**Authors:** Yumeng Liu, Bin Zhao

**Affiliations:** 1 Department of Building Science, School of Architecture, Tsinghua University, Beijing, China; 2 Beijing Key Laboratory of Indoor Air Quality Evaluation and Control, Tsinghua University, Beijing, China

*To the Editor*—A recent article by Liu et al^1^ reports that severe acute respiratory syndrome coronavirus 2 (SARS-CoV-2) was detected in the air in hospital areas occupied by inpatients, medical staff, and the public. Their study reported the size distribution of SARS-CoV-2–laden aerosols, which makes it possible to evaluate the filtration efficiencies required for face masks and respirators. The use of face masks and respirators has been recommended for protecting healthcare workers and the general public during the COVID-19 pandemic because SARS-CoV-2 is widely distributed in the air of Wuhan hospitals^2,3^ and the virus can remain viable in aerosols for several hours.^4^ However, the size-dependent filtration efficiencies of face masks and respirators for removing SARS-CoV-2–laden aerosols have not yet been reported.

**Fig. 1.**
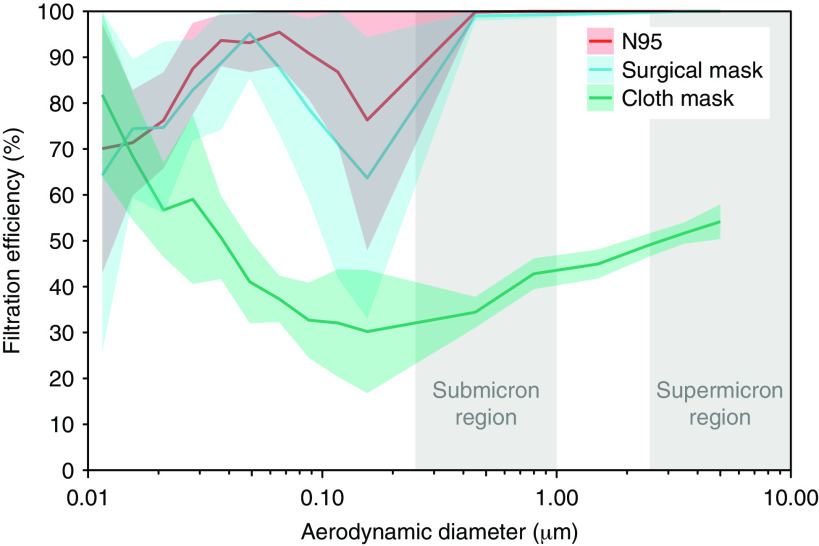
Filtration efficiencies of an N95 respirator, a surgical mask, and a cloth mask. The filtration efficiencies are combined from Konda et al.^5^ The cloth mask is made of double-layered quilter’s cotton with a thread-count of 80 threads per inch, which is often used in do-it-yourself masks. The flow rate was 1.2 cubic feet per minute (CFM), which represents human respiration rates at rest. The graph lines represent the mean values, and the shaded areas represent the standard error of 7 experiments.

The study by Liu et al^1^ reported that SARS-CoV-2 occurs most commonly in aerosols in the submicron (0.25–1.0 μm) and supermicron (>2.5 μm) ranges. We reviewed the measured filtration efficiencies of different masks and respirators reported by Konda et al^5^ and compared them to sizes of SARS-CoV-2–laden aerosols (Fig. 1). The N95 respirator and surgical mask show a high filtration efficiency of >60% for aerosols in the submicron range and almost 100% for aerosols in the supermicron range. The filtration efficiency of cloth masks is much lower (<50%). Therefore, for healthcare workers facing a high risk of opportunistic airborne infection, an N95 respirator or surgical mask is sufficient to defend against SARS-CoV-2. For people at low risk of infection, a homemade cloth face mask is a suitable alternative if there are shortages of face masks and respirators, provided that the correct fabrics are used.^5^ Importantly, any leakage from the edges of the face mask may result in a significant decrease in filtration efficiency,^6^ so face masks must fit correctly to be effective.

With the spread of the COVID-19 pandemic, numerous findings have shown the protective effect of wearing face masks,^7^ which increases public recognition of the need for them. Considering the limited supplies of face masks, the rational use different types of face masks or respirators depending on the pandemic severity and context of use has been advised.^8^ In the meantime, the size-dependent filtration efficiencies for SARS-CoV-2–laden aerosols reported here can be used to inform policies on the use of face masks.
